# Appetite disinhibition rather than hunger explains genetic effects on adult BMI trajectory

**DOI:** 10.1038/s41366-020-00735-9

**Published:** 2021-01-14

**Authors:** Eric J. Brunner, Koutatsu Maruyama, Martin Shipley, Noriko Cable, Hiroyasu Iso, Ayako Hiyoshi, Daryth Stallone, Meena Kumari, Adam Tabak, Archana Singh-Manoux, John Wilson, Claudia Langenberg, Nick Wareham, David Boniface, Aroon Hingorani, Mika Kivimäki, Clare Llewellyn

**Affiliations:** 1 Institute of Epidemiology and Health Care, University College London, London, UK; 2 Public Health, Department of Social Medicine, Osaka University Graduate School of Medicine, Suita, Japan; 3 Laboratory of Community Health and Nutrition, Department of Bioscience, Graduate School of Agriculture, Ehime University, Matsuyama, Japan; 4 Clinical Epidemiology and Biostatistics, School of Medical Sciences, Örebro University, Örebro, Sweden; 5 Nutrepol, Merritt Island, FL USA; 6 Institute for Social and Economic Research, University of Essex, Colchester, UK; 7 Department of Internal Medicine and Ocology, Faculty of Medicine, Semmelweis University, Budapest, Hungary; 8 Department of Public Health, Faculty of Medicine, Semmelweis University, Budapest, Hungary; 9 Epidemiology of Ageing and Neurodegenerative diseases, Université de Paris, Paris, France; 10 North Devon Medical Education Centre, North Devon District Hospital, Barnstaple, UK; 11 MRC Epidemiology Unit, Institute of Metabolic Science, University of Cambridge, Cambridge, UK; 12 Institute of Cardiovascular Science, University College London, London, UK

**Keywords:** Lifestyle modification, Epidemiology

## Abstract

**Background/objectives:**

The mediating role of eating behaviors in genetic susceptibility to weight gain during mid-adult life is not fully understood. This longitudinal study aims to help us understand contributions of genetic susceptibility and appetite to weight gain.

**Subjects/methods:**

We followed the body-mass index (BMI) trajectories of 2464 adults from 45 to 65 years of age by measuring weight and height on four occasions at 5-year intervals. Genetic risk of obesity (gene risk score: GRS) was ascertained, comprising 92 BMI-associated single-nucleotide polymorphisms and split at a median (=high and low risk). At the baseline, the Eating Inventory was used to assess appetite-related traits of ‘disinhibition’, indicative of opportunistic eating or overeating and ‘hunger’ which is susceptibility to/ability to cope with the sensation of hunger. Roles of the GRS and two appetite-related scores for BMI trajectories were examined using a mixed model adjusted for the cohort effect and sex.

**Results:**

Disinhibition was associated with higher BMI (*β* = 2.96; 95% CI: 2.66–3.25 kg/m^2^), and accounted for 34% of the genetically-linked BMI difference at age 45. Hunger was also associated with higher BMI (*β* = 1.20; 0.82–1.59 kg/m^2^) during mid-life and slightly steeper weight gain, but did not attenuate the effect of disinhibition.

**Conclusions:**

Appetite disinhibition is most likely to be a defining characteristic of genetic susceptibility to obesity. High levels of appetite disinhibition, rather than hunger, may underlie genetic vulnerability to obesogenic environments in two-thirds of the population of European ancestry.

## Introduction

Obesity is a global health problem [1, 2] and the modern environment contributes importantly to this epidemic [3–5]. Little is known about individual susceptibility to the obesogenic environment. Adiposity is partly heritable [6] and many adiposity-associated genetic variants have been identified [7]. High genetic risk of obesity is manifested among children, linked with large appetite, eating in the absence of hunger, [8] a weak sense of satiety [9, 10], and high energy intake [11]. Even in early infancy, very young children with a larger appetite gain weight faster [12–14]. In adults, elevated genetic risk of obesity is also associated with appetite, blunted satiety [15], and high responsiveness to external food cues [16–19]. However, it is not known whether genetic risk of weight gain continues to operate via appetite mechanisms during adulthood because there are no longitudinal studies of genetic risk and appetite-related phenotypes.

We conducted a longitudinal study to identify the roles of genetic risk of obesity, appetite-related traits for the 20-year weight gain during mid-life. We examined clinically assessed body-mass index (BMI) trajectories in men and women from age 45 to 65 with the UK civil service, i.e., Whitehall II cohort according to gene risk score, and two appetite-related traits, using a multi-item eating behavior questionnaire [20].

## Subjects and methods

### Study population

Individuals were recruited to the Whitehall II cohort study in 1985–1988 from civil servants working in the London offices of 20 Whitehall departments in London [21]. 10,308 individuals participated, with a response rate of 73%. In 1997–1999, the baseline for this study, 6551 individuals participated in the research clinic for medical check-up. At the 2003–2004 clinic, participants of European ancestry provided blood, which was used for genotyping BMI-associated SNPs. We analyzed 1896 men and 568 women aged 45–65 years at baseline with complete data available on genes, eating behaviors and BMI (collected at clinic phases 5, 7, 9, and 11, 1997–2013; see Fig. S1). The University College London Research Ethics Committee approved the study. Participants gave informed consent at each data collection.

### Appetite-related trait assessment

Two appetite-related traits, hunger and disinhibition, were assessed through self-report responses to Stunkard and Messick’s Eating Inventory (EI), administered at baseline [20]. Hunger is defined as the internal physiological urge or drive to eat, relating to susceptibility to and ability to cope with the sensation of hunger [22]. Appetite disinhibition is defined as the opportunistic eating or overeating response to environmental and emotional cues. Disinhibition may involve several behavioral traits: high responsiveness to external food cues e.g., sight of attractive foods on display in a shop, smelling hot, freshly-prepared foods; eating in the absence of hunger; eating in an uncontrolled way (binge eating); over-eating in social situations. Emotionally cued eating concerns eating in response to low mood, depression or anxiety; eating as a response to loneliness.

The EI includes assessment of disinhibition and hunger with good reliability and validity (see Supplementary Text) [20]. Internal consistency of the disinhibition and hunger scales was evaluated using Cronbach’s alpha in the present sample (Disinhibition: men 0.77, women 0.84; hunger: men 0.72, women 0.76). Disinhibition (16 items). Hunger (14 items). Up to one missing item response per subscale was allowed, scaling up the score proportionally. Among the 6551 individuals who participated at the clinic screening in 1997–1999, the EI was administered to 5308 (81%) (Fig. S1). Among those of European ethnicity (*N* = 4925), the EI was fully completed and appetite traits ascertained for 4794 (97%) participants.

### Adiposity measurement

BMI was calculated as weight(kg)/height(m)^2^ based on clinic measurements following standard protocols [21]. BMI was assessed four times at 5-year intervals (1997–99, 2002–04, 2007–09 and 2012–2013). Among the 2464 participants, the percentage of participants who had BMI was 88% in 1997–99, 99% in 2002–04, 92% in 2007–09, and 84% in 2012–2013, respectively (Fig. S1). We also identified maximum BMI during follow-up, for the purpose of a sensitivity analysis (see Statistical Analysis section for detail).

### Gene risk score

DNA was extracted from blood samples collected in the 2003–2004 phase 7 clinic using magnetic bead technology. Genotyping with the metabochip, a custom Illumina iSelect genotyping array, was successful for 5441 participants (78.7% of 6914 phase 7 participants) of whom 5067 reported European ethnicity [23]. We measured 92 of 97 independent obesity-related SNPs previously associated with BMI in GWAS [7]. The genotype frequencies did not deviate from Hardy–Weinberg equilibrium (*p* > 0.05). We calculated a genetic risk score (GRS) for all available BMI-related loci on each individual, based on an additive model of genetic risk. We split the GRS at the median (high vs. low).

### Covariates

Age and sex were reported by participants. For all analyses, age was centered at 60 years. Birth year was centered at 1940 to take account of the secular trend in BMI changes [24].

### Study design overview

We used longitudinal modelling to analyze trajectories of weight gain between ages 45 and 65 years with BMI as outcome (dependent variable). After age 65, mean BMI plateaued and then tended to decline. Three risk factors were examined separately in relation to age 45 (baseline) BMI and BMI change: (1) gene risk score (2) hunger, and (3) disinhibition. For the second part of the study we conducted mediation analysis to compare the extent to which the two appetite traits (hunger, disinhibition) accounted statistically for the effect of gene risk score on baseline BMI and BMI trajectory.

### Statistical analysis

Linear mixed effect models were fitted to examine heterogeneity in BMI trajectories by age, taking into account effects of sex, year of birth and linear and curvilinear (quadratic) effects over the follow-up. To examine BMI trajectories according to GRS and appetite-related traits we dichotomized the scores as either low or high according to the median values using the maximum number of participants available. The effect of GRS and appetite-related traits on BMI trajectories was examined by fitting the interaction of the linear term for age with each of the dichotomized exposures of interest. The estimates from these models were used to plot BMI trajectories for participants with low and high GRS and appetite-related traits.

After that, the effects of the appetite-related traits on the association of GRS with BMI trajectory intercept were estimated using the equation ((β1 – β0)/β0) × 100%, where β0 is the coefficient for GRS in a model with covariates and β1 is the coefficient for GRS in a model with covariates + continuous appetite-related score. We calculated 95% CI for the attenuations, i.e., mediation using the bootstrap method with 2000 re-samplings to examine the role of appetite traits.

Further, we examined the joint effect of the two appetite traits by creating a variable with three levels according to the number of times (0, 1 or 2) participants were in the high category for the two appetite traits (Supplementary Text). Also, using just one observation per participant, we estimated the effects of appetite-related traits on the associations of GRS with the maximum BMI attained during follow-up using multiple regression and used the bootstrap method with 2000 re-samplings to calculate 95% CI for these attenuations as a sensitivity analysis (Supplementary files). Probability values for statistical tests were 2-tailed. All analyses were conducted using STATA 15.1 (StataCorp, College Station, TX, USA).

## Results

Genetic data were available, and GRS known on 2464 (51%) of the participants who completed the EI. A greater proportion of those with known, compared to unknown, GRS were men (77% vs. 68%) and they were 0.8 years younger (*p* < 0.001), but baseline BMI did not differ between groups (both 26.1 kg/m^2^, *p* = 0.65). Mean baseline BMI, disinhibition and hunger was 25.7 kg/m^2^, 4.38 and 3.37 in the low genetic risk group and 26.6 kg/m^2^, 4.95 and 3.62 in the high-risk group (Table 1). There were larger proportions of participants with high scores for disinhibition and hunger in the high (55.3%, 42.5%, respectively) compared to the low genetic risk group (48.3%, 38.2% respectively).

**Table 1:** Baseline characteristics by gene risk score for adiposity.

	Gene risk score				
	Low		High		*P* value
Number of participants	1329		1135		
Men (%)	78.4		75.2		0.063
Age, Mean (SD)	55.4	(6.0)	55.5	(6.0)	0.82
BMI, Mean (SD)	25.7	(3.7)	26.6	(3.8)	<0.0001
Gene risk score, Mean (SD)	84.5	(3.9)	94.1	(3.6)	<0.0001
Disinhibition, Mean (SD)	4.38	(3.11)	4.95	(3.42)	<0.0001
High disinhibition (%)	48.3		55.3		0.0005
Hunger, Mean (SD)	3.37	(2.59)	3.62	(2.73)	0.018
High hunger (%)	38.2		42.5		0.032

High categories defined as being above the median score in the whole cohort.

**Fig. 1:**
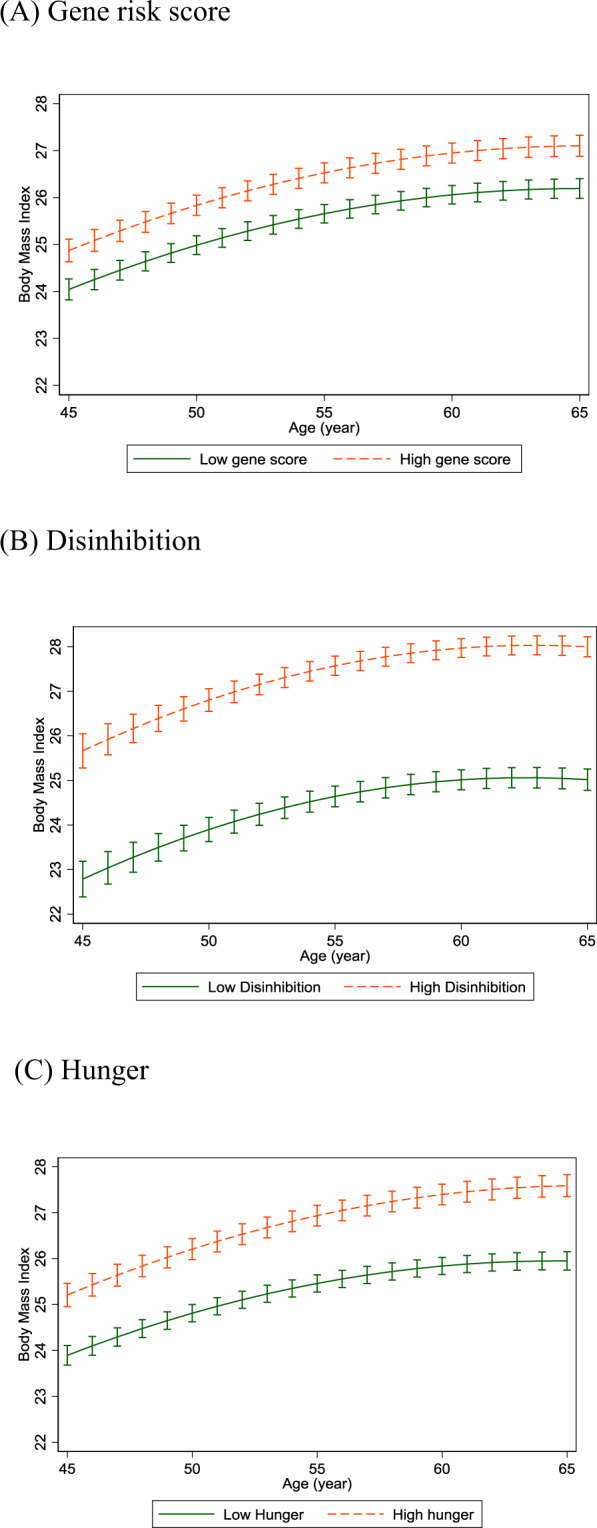
BMI trajectories to age 65, predictions from longitudinal model with gene risk score and each eating behavior. (*N* = 2464). Error bars show 95% CI around fixed effects. Orange dashed line indicates those with gene risk score or eating behavior above median; Green solid line indicates those with gene risk score or eating behavior below median. BMI values are adjusted for sex and birth cohort effects.

Table 2 and Fig. 1 show the BMI trajectories from age 45 to 65 years, predicted with linear mixed models of genetic risk and appetite traits. On average, BMI increased by 2.1 kg/m^2^ in men and 2.6 kg/m^2^ in women during the observation; 6.1 kg (13 lb) and 7.5 kg (17 lb) respectively for an adult 1.7 m (5′7′′) tall. BMI trajectories were curvilinear and increased in parallel to age 65 for high and low levels of genetic risk (Fig. 1A; age interaction *p* = 0.54).

**Table 2:** Coefficients from linear mixed models of BMI trajectory comparing high^a^ vs. low gene risk score and eating behaviors (disinhibition and hunger) and their interactions with age, for BMI measurements at ages 45–65 years. (*N* = 2464).

	Coefficient^b^	(95% CI)	*P* value
High gene risk score^c^	0.88	(0.56, 1.20)	<0.0001
Interaction: High gene risk score × age	0.005	(−0.010, 0.019)	0.54
High disinhibition^c^	2.96	(2.66, 3.25)	<0.0001
Interaction: High disinhibition × age	0.005	(−0.009, 0.020)	0.49
High hunger^c^	1.20	(0.82, 1.59)	<0.0001
Interaction: High hunger × age	0.023	(0.008, 0.038)	0.002

^a^High categories defined as being above the median score in the whole cohort.

^b^Coefficients are adjusted for sex, linear (age) and curvilinear (age squared) trend and birth cohort effect on BMI trajectories.

^c^Effects of high gene risk score, disinhibition and hunger shown for age 45.

**Fig. 2:**
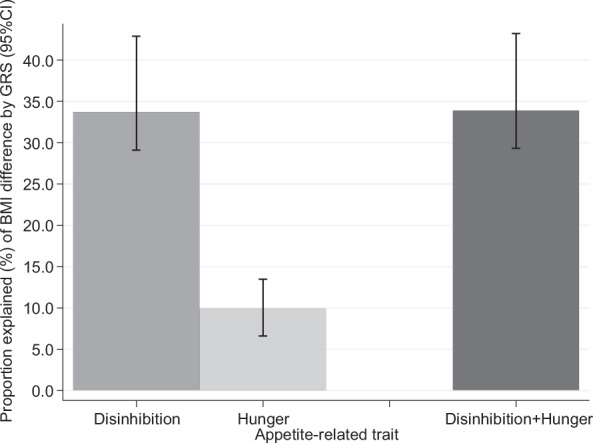
Mediation analysis of gene risk score difference in BMI by appetite-related traits in adults aged 45–65 years. (*N* = 2464). The proportion of BMI differences according to genetic risk score explained by disinhibition and hunger was estimated by adding each appetite-related trait to a model for BMI, separately and simultaneously. The 95% confidence intervals for the mediation proportions were estimated using a bootstrap procedure. The model was adjusted for sex, linear and curvilinear age (age^2^), birth cohort effect on BMI, and the eating behavior *age interaction. Disinhibition level accounted for 33.7% (95% confidence interval 29.1–42.9%) of the GRS-BMI association.

We found that high genetic risk was associated with higher average BMI from 45 to 65 years (difference 0.88 kg/m^2^). In regard to appetite traits, the high disinhibition group had substantially higher average BMI from 45 to 65 years (difference 2.96 kg/m^2^). BMI trajectories increased in parallel in the high and low disinhibition groups (Fig. 1B; age interaction *p* = 0.49). High hunger was also associated with higher BMI, but relatively smaller increase (difference 1.20 kg/m^2^) at age 45. BMI increased faster in the high hunger group (0.023 kg/m^2^ larger increase in BMI per year over follow-up) compared to the low hunger group, between ages 45 and 65 (Fig. 1C; age interaction *p* = 0.0021). The results were unchanged in a mutually adjusted model that included disinhibition and hunger scores together, and when using BMI measurements at all ages >45 years (Table S1). We examined the extent to which appetite-related traits accounted for the association between GRS and average BMI from 45 to 65 years of age, applying mediation analysis (Table S2). Disinhibition level accounted for 33.7% (95% confidence interval 29.1–42.9%) of the GRS-BMI association (Fig. 2). There was no further attenuation when hunger was added into the model, indicating that the association between genetic risk and average BMI in mid-life was mediated only by disinhibition.

The joint effect of appetite traits model also confirmed that the combination of high hunger and high disinhibition (0, 1, 2) and BMI trajectories were associated with the highest and steepest BMI trajectories from age 45 to 65 (interaction between appetite combination group and age, *p* < 0.0001) (Fig. S2). Results in the sensitivity analyses were similar using measures of BMI at all ages >45 years (Table S3) and using maximum BMI, supporting the attenuating role of the appetite disinhibition in relation to GRS and BMI (Table S4).

## Discussion

BMI increased during mid-life, with an average 20-year gain to age 65 of 2.1 and 2.6 kg/m^2^ in men and women respectively. The associations between genetic risk, disinhibition and BMI were established age 45. Disinhibited eating was a defining characteristic of adults with higher BMI throughout mid-life, and of those with higher genetic susceptibility to adiposity.

Among those scoring above the median on disinhibition, average BMI was nearly 3.0 units higher than those with low disinhibition, equating to a difference of 8.6 kg (or 19 lbs.) for an adult of average height (1.7 m). Disinhibition accounted for approximately one-third of the association between genetic susceptibility to obesity and average BMI during mid-life. We observed that high hunger was associated with higher BMI at age 45 and BMI increased faster in the high hunger group compared to the low hunger group. Nevertheless, hunger had no significant and independent explanatory effect in our mediation analysis. Based on our findings, we think appetite disinhibition—overeating in response to external food cues—is a focal behavior underpinning genetic variation in susceptibility to the obesogenic environment.

### Findings in context

Disinhibited or uncontrolled eating is a characteristic of adults at higher genetic risk of obesity [16–19]. Behavioral studies have shown aberrations in neurobehavioral markers of appetite, including the tendency to eat in the absence of hunger, and hyper-responsiveness to food cues among children [8, 11] and adults [25] according to genetic susceptibility to obesity. Gene expression studies support that many BMI-linked common genetic variants are expressed in brain areas with appetite regulation functions [7].

There is consistent longitudinal evidence from birth cohort studies that genetic risk of obesity is linked with weight gain throughout infancy and childhood [26–28]. Previous findings showed that the length of the period of genetic effects in early adult life was likely subject to considerable individual variation [29–31]. However, in our study, a difference in mean BMI of 0.88 kg/m^2^ between high and low genetic risk groups was established even after age 45 and it was stable at the age group level over the subsequent two decades.

Our statistical mediation models provide strongly that the level of disinhibition of appetite in response to environmental stimulus helps in understanding the nature of the global epidemic of obesity. The individual disinhibition score mediated around one-third of the genetic association with adiposity. On the other hand, the score on the internal physiological hunger trait scale, corresponding to the intrinsic urge to eat, accounted for none of that association in the mediation model.

Disinhibition is a tendency to want to eat in response to the sight, smell or taste of palatable food [20]. Its expression is likely strongly dependent on the food environment, and the current ubiquity of external food cues provides the ideal context for full expression of this trait. The link between appetite disinhibition and adiposity is congruent with findings in infants and children [12–14], to which our findings offer extensive support. The disinhibition scale also measures the extent to which emotional reactions lead to overeating [20]. Several studies have shown obesity-related risk alleles were associated with emotional eating or emotional problems [32, 33], but the findings are controversial. Therefore, emotional eating might partly explain the association between obesity-related risk alleles and BMI trajectory.

Hunger, in contrast to the disinhibition component of appetite, is the drive to eat governed by internal neurobehavioral processes, as distinct from the desire to eat in response to external food cues [20, 22]. Eating behaviors exhibit long-term stability [34, 35]; we also observed that higher hunger scores were associated with higher BMI in mid-life and slightly greater weight gain equating to 0.5 BMI units over 20 years from age 45. Nevertheless, we did not find the significant role played by hunger compared to disinhibition. The declining rate of weight gain during later adulthood in our findings suggests that hunger may decline incrementally from mid-life to older ages.

Longitudinal data on genetic influences on clinically measured BMI trajectories from mid-life to older age are rare [29–31, 36, 37], and there have been no longitudinal studies to date incorporating appetite-related traits in mid-life [38–43]. The measured appetite traits, mainly disinhibition accounted moderately. Although appetite disinbition did not account fully for genetic susceptibility to high BMI, our findings point the potential relevance of appetite-related traits such as satiety sensitivity.

### Strengths and limitations

We used a well-validated instrument to assess eating inventories in our study sample. BMI was measured on four occasions by a research nurse adhering to a standard protocol. With 20 years of longitudinal observation we were able to assess how gene risk score and eating inventories were independently and jointly related to BMI trajectory in a large sample of ethnically white adults. Among 2464 participants, the proportion with a BMI measurement was high at each phase (Fig S1). The high participation rate, with little drop-out over the two decades of the study, indicates that the BMI trajectories are subject to little health selection bias. However, the EI was administered only at baseline and we could not take behavioral change into account. It would be useful to extend this line of research to examine the dynamic associations between genetic risk, eating behavior, and weight change.

Genotyping was based on DNA collected at a later clinic phase than the study baseline (Fig S1) and the sample size was reduced because genotype data were missing. Potential selection bias was assessed by comparing cross-sectional correlations of disinhibition and hunger scores with BMI, and age trajectories of BMI, between those in the study sample and those excluded (Fig S3). The correlation coefficient between disinhibition and BMI was 0.46 in the study sample and *r* = 0.41 in those excluded (difference *p* = 0.08). The respective coefficients for hunger were 0.24 and 0.22 (difference *p* = 0.50). The lack of difference in these associations between those with and without GRS suggests the main associations of interest are not biased by the incomplete genotype data.

Whitehall II is an occupational cohort of British civil servants [22]. We consider that the associations between obesity-related genes and BMI trajectories, and the role of appetite traits in mediating the gene-BMI association is not substantially different from the general population. However, replication studies, including children and young adults as well as older adults, of the independent and joint effects of obesity-related genes and appetite disinhibition in relation to BMI trajectories would be useful.

### Implications

Overweight and obesity can be a result from a combination of genetic susceptibility to overeating and exposure to an appetite facilitating food environment, i.e., food cues. Changes to the environment to reduce the frequency and intensity of food cues may have a positive impact in a population, shifting the distribution of BMI to a lower level. Among individuals, guidance about healthy food choices is important. It could be enhanced by emphasizing the particular value for disinhibited eaters of avoiding cues to overeat. Such cues may be direct, e.g., food on display, or indirect signals of food availability e.g., retail stores and product advertisements. Experimental studies show that such indirect cues increase motivation to actively seek out and consume food in the absence of hunger [44].

For individuals, the predictive value of genetic risk information for obesity is modest. Further, our findings suggest that appetite, especially disinhibition, is an important marker of obesity risk providing a pointer to tailored intervention. Measuring disinhibition and identifying problematic eating behaviors and environmental triggers could allow clinical advisors to support their patients to develop strategies to manage eating behavior and achieve weight-loss goals.

## Conclusion

Appetite disinhibition appears to be a key characteristic, for adults at high genetic risk of obesity, given the epidemic of excessive weight gain. The trait of low appetite disinhibition may provide a clue to the elusive reason why some adults remain lean, despite exposure to obesogenic environments, to which so many are susceptible. Modification of the wider food environment is likely to be a valuable public health endeavor. Our findings suggest a potential clinical strategy which includes support for individuals with high appetite disinhibition to avoid food cues, in addition to their genetic risk status.

## Supplementary information

SUPPLEMENTARY INFORMATION
