# Correction: Appetite disinhibition rather than hunger explains genetic effects on adult BMI trajectory

**DOI:** 10.1038/s41366-021-00770-0

**Published:** 2021-02-01

**Authors:** Eric J. Brunner, Koutatsu Maruyama, Martin Shipley, Noriko Cable, Hiroyasu Iso, Ayako Hiyoshi, Daryth Stallone, Meena Kumari, Adam Tabak, Archana Singh-Manoux, John Wilson, Claudia Langenberg, Nick Wareham, David Boniface, Aroon Hingorani, Mika Kivimäki, Clare Llewellyn

**Affiliations:** 1 Institute of Epidemiology and Health Care, University College London, London, UK; 2 Public Health, Department of Social Medicine, Osaka University Graduate School of Medicine, Suita, Japan; 3 Laboratory of Community Health and Nutrition, Department of Bioscience, Graduate School of Agriculture, Ehime University, Matsuyama, Japan; 4 Clinical Epidemiology and Biostatistics, School of Medical Sciences, Örebro University, Örebro, Sweden; 5 Nutrepol, Merritt Island, FL USA; 6 Institute for Social and Economic Research, University of Essex, Colchester, UK; 7 Department of Internal Medicine and Ocology, Faculty of Medicine, Semmelweis University, Budapest, Hungary; 8 Department of Public Health, Faculty of Medicine, Semmelweis University, Budapest, Hungary; 9 Epidemiology of Ageing and Neurodegenerative diseases, Université de Paris, Paris, France; 10 North Devon Medical Education Centre, North Devon District Hospital, Barnstaple, UK; 11 MRC Epidemiology Unit, Institute of Metabolic Science, University of Cambridge, Cambridge, UK; 12 Institute of Cardiovascular Science, University College London, London, UK

**Keywords:** Lifestyle modification, Epidemiology

Correction to: *International Journal of Obesity*

10.1038/s41366-020-00735-9

The original version of this article unfortunately contained a mistake in the ESM. The original article has been corrected.

## Supplementary information

Supplementary Information

