# Supplementary material for: Appetite disinhibition rather than hunger explains genetic effects on adult BMI trajectory
Source: Int J Obes (Lond). 2021 Jan 14;45(4):758–65. doi: 10.1038/s41366-020-00735-9 (PMC8005371; doi:10.1038/s41366-020-00735-9)
Supplement: Supplementary file 1 — SUPPLEMENTARY INFORMATION [file 41366_2020_735_MOESM1_ESM.docx]

SUPPLEMENTARY INFORMATION

TEXT

Stunkard-Messick Eating Inventory (p.2)

FIGURES

Fig S1. Selection of analytic sample and BMI measurements obtained (p.5)

Fig S2. BMI trajectories to age 65, predictions from longitudinal model showing the joint effects of disinhibition (D) and hunger (H). (N=2464) (p.6)

Fig S3. BMI trajectories to age 65, predictions from longitudinal model in analytic samples and the samples who did not have genetic data (p.7)

TABLES

Table S1. Coefficients from linear mixed models of BMI trajectory comparing higha versus low, gene risk score and eating behaviours (disinhibition and hunger) and their interactions with age, for BMI measurements at all ages >45 years. (N=2464) (p.8)

Table S2. Effect of high versus low gene risk score (GRS) on BMI trajectory during follow-up and attenuation on adjustment for appetite traits, for BMI measurements at ages 45-65 years. (N=2464) (p.9)

Table S3. Effect of high versus low gene risk score (GRS) on BMI trajectory during follow-up and attenuation on adjustment for appetite traits, for BMI measurements at all ages ≥45 years. (N=2464) (p.10)

Table S4: Effect of high, versus low gene risk score on maximum BMI during follow-up and attenuation on adjustment for appetite traits. (N=2464) (p.11)

TEXT

Stunkard-Messick Eating Inventory (reference 20 for the main text)

Disinhibition and hunger subscales for the Eating Inventory are shown separately below. Items are mixed together in the questionnaire as used. See Methods section of article for scoring system.

--

Please read each of the following 36 statements carefully. If you agree with the statement, or feel that it is trues applied to you, answer true by blocking in the appropriate rectangle like this ■. If you disagree with the statement, or feel that it is false as applied to you, answer false by blocking in the appropriate rectangle.

Disinhibition

1. When I smell a roasting chicken or see a juicy piece of meat, I find it very difficult to keep from eating, even if I have just finished a meal. True □ False □
2. I usually eat too much at social occasions, like parties and picnics. True □ False □
3. Sometimes things just taste so good that I keep on eating even when I am no longer hungry. True □ False □
4. When I feel anxious, I find myself eating. True □ False □
5. Since my weight goes up and down, I have gone on reducing diets more than once. True □ False □
6. When I am with someone who is overeating, I usually overeat too. True □ False □
7. Sometimes when I start eating, I just can't seem to stop. True □ False □
8. It is not difficult for me to leave something on my plate. True □ False □
9. When I feel low, I often overeat. True □ False □
10. My weight has hardly changed at all in the last ten years. True □ False □
11. When I feel lonely, I console myself by eating. True □ False □
12. Without even thinking about it, I take a long time to eat. True □ False □
13. While on a diet, if I eat a food that is not allowed. I often then 'splurge' and eat other high calorie food. True □ False □

Each question in this section is followed by a number of answer options. After reading each question carefully, choose the one option which most applies to you, and block in the appropriate rectangle.

1. Do you eat sensibly in front of others and 'splurge' alone?

Never □ Rarely □ Often □ Always □

1. Do you go on eating binges even though you are not hungry?

Never □ Rarely □ Sometimes □ At least once a week □

1. To what extent does this statement describe your eating behaviour? “I start dieting in the morning, but because of any number of things that happen during the day, by evening I have given up and eat what I want, promising myself to start dieting again tomorrow."

Not like me □ A little like me □ Pretty good description of me □ Describes me perfectly □

Hunger

1. I am usually so hungry that I eat more than three times a day. True □ False □
2. Dieting is so hard for me because I just get too hungry. True □ False □
3. Since I am often hungry, I sometimes wish that while I am eating, an expert would tell me that I have had enough or that I can have something more to eat. True □ False □
4. I often feel so hungry that I just have to eat something. True □ False □
5. At certain times of the day, I get hungry because I have become used to eating then. True □ False □
6. Being with someone who is eating often makes me hungry enough to eat also. True □ False □
7. When I see a real delicacy, I often get so hungry that I have to eat right away. True □ False □
8. I get so hungry that my stomach often seems like a bottomless pit. True □ False □
9. I am always hungry so it is hard for me to stop eating before I finish the food on my plate.

True □ False □

1. l sometimes get very hungry late in the evening or at night. True □ False □
2. I am always hungry enough to eat at any time. True □ False □

Each question in this section is followed by a number of answer options. After reading each question carefully, choose the one option which most applies to you, and block in the appropriate rectangle.

1. How often do you feel hungry?

Only sometimes □ sometimes between meals □ Often between meals □ Almost always □

1. How difficult would it be for you to stop eating halfway through dinner and not eat for the next four hours?

Easy □ Slightly difficult □ Moderately difficult □Very difficult □

1. How frequently do you skip dessert because you are no longer hungry?

Almost never □ Seldom □ At least once a week □ Almost every day □

Fig S1.

Selection of analytic sample and BMI measurements obtained

10,308

306 deaths: Phase 1 to phase 5
2,132 no response at Phase 5

1,319 participated at Phase 5 but did not

attend screening clinic

Genotype data (phase 7)

2330 gene risk score missing.

2,562 no Eating Inventory data at Phase 5

2,464

4,794

131 one or both appetite traits incomplete

383 not white ethnicity

4,925

6,551

5,308

Phase 7 BMI

2,458 (98.8%)

Phase 5 BMI

2,171 (88.1%)

Phase 9 BMI

2,259 (91.7%)

Phase 11 BMI

2,070 (84.0%)

Fig S2.

BMI trajectories to age 65, predictions from longitudinal model showing the joint effects of disinhibition (D) and hunger (H). (N=2464)


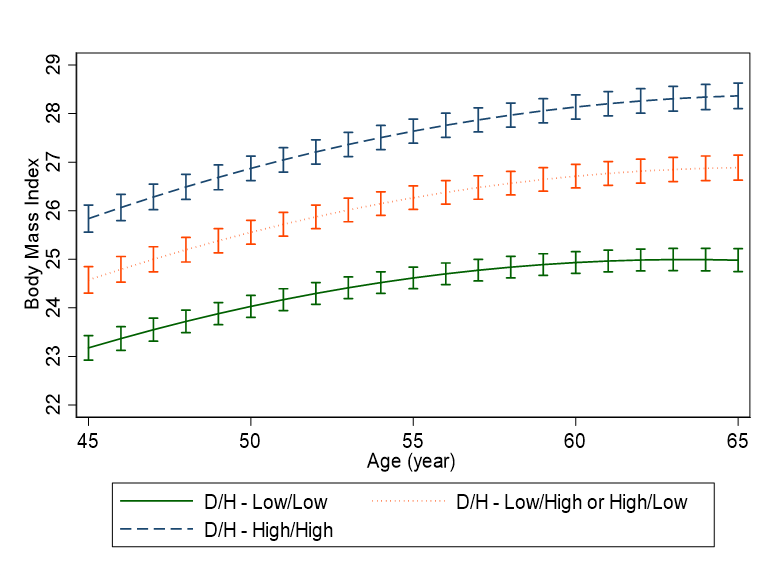


Fig S3.

BMI trajectories to age 65, predictions from longitudinal model in analytic samples and the samples who did not have genetic data.

The trajectories did not differ in the two groups (p for interaction with age = 0.37)

Table S1.

Coefficients from linear mixed models of BMI trajectory comparing high^a^ versus low, gene risk score and eating behaviours (disinhibition and hunger) and their interactions with age, for BMI measurements at all ages >45 years. (N=2464)

|  | Coefficient^a^ | (95%CI) | P-value |
| --- | --- | --- | --- |
| High gene risk score^c^ | 0.90 | (0.59, 1.21) | <0.0001 |
| Interaction: High gene risk score x age (Model 1^b^) | -0.001 | (-0.011, 0.009) | 0.84 |
|  |  |  |  |
| High disinhibition^c^ | 2.97 | (2.68, 3.26) | <0.0001 |
| Interaction: High disinhibition x age (Model 1^b^) | 0.002 | (-0.008, 0.012) | 0.74 |
|  |  |  |  |
| High hunger^c^ | 1.39 | (1.02, 1.75) | <0.0001 |
| Interaction: High hunger x age (Model 1^b^) | 0.014 | (0.004, 0.024) | 0.0061 |

^a^ High categories defined as being above the median score in the whole cohort

^b^ Model 1 coefficients are adjusted for sex, linear (age) and curvilinear (age squared) trend and birth cohort effect on BMI trajectories

^c^ Effects of high gene risk score, disinhibition and hunger shown for age 45

Table S2.

Effect of high versus low gene risk score (GRS) on BMI trajectory during follow-up and attenuation on adjustment for appetite traits, for BMI measurements at ages 45-65 years. (N=2464)

| Model | High vs. low GRS | | |  | Attenuation |
| --- | --- | --- | --- | --- | --- |
|  | Coefficient | (95%CI) | P-value |  | Percent (95% CI) |
|  |  |  |  |  |  |
| Base model^a^ | 0.88 | (0.56, 1.19) | <0.0001 |  | Reference |
| Base model + Disinhibition | 0.59 | (0.30, 0.86) | <0.0001 |  | 33.7 (29.1, 42.9) |
| Base model + Hunger | 0.80 | (0.48, 1.10) | <0.0001 |  | 9.9 (6.6, 13.5) |
|  |  |  |  |  |  |
| Base model + both appetite traits | 0.59 | (0.30, 0.86) | <0.0001 |  | 33.9 (29.3, 43.2) |
|  |  |  |  |  |  |

^a^ Base model is adjusted for sex, linear (age) and curvilinear (age squared) trend and year of birth

**Table S3.**

Effect of high versus low gene risk score (GRS) on BMI trajectory during follow-up and attenuation on adjustment for appetite traits, for BMI measurements at all ages ≥45 years. (N=2464)

| Model | High vs. low GRS | | |  | Attenuation |
| --- | --- | --- | --- | --- | --- |
|  | Coefficient | (95%CI) | P-value |  | Percent (95% CI) |
|  |  |  |  |  |  |
| Base model^a^ | 0.90 | (0.59, 1.21) | <0.0001 |  | Reference |
| Base model + Disinhibition | 0.60 | (0.32, 0.87) | <0.0001 |  | 33.8 (30.4, 37.4) |
| Base model + Hunger | 0.81 | (0.51, 1.11) | <0.0001 |  | 10.1 (8.4, 11.9) |
|  |  |  |  |  |  |
| Base model + both appetite traits | 0.59 | (0.32, 0.87) | <0.0001 |  | 34.0 (30.5, 37.9) |
|  |  |  |  |  |  |

^a^ Base model is adjusted for sex, linear (age) and curvilinear (age squared) trend and year of birth

**Table S4:**

Effect of high, versus low gene risk score on maximum BMI during follow-up and attenuation on adjustment for appetite traits. (N=2464)

| Model | High vs. low GRS | | |  | Attenuation |
| --- | --- | --- | --- | --- | --- |
|  | Coefficient | (95%CI) | P-value |  | Percent (95% CI) |
|  |  |  |  |  |  |
| Base model^a^ | 0.99 | (0.65, 1.33) | <0.0001 |  | Reference |
| Base model + Disinhibition | 0.65 | (0.35, 0.96) | <0.0001 |  | 34.1 (19.2, 53.4) |
| Base model + Hunger | 0.90 | (0.55, 1.22) | <0.0001 |  | 10.9 (2.4, 22.0) |
|  |  |  |  |  |  |
| Base model + both appetite traits | 0.65 | (0.34, 0.95) | <0.0001 |  | 34.5 (19.7, 53.1) |
|  |  |  |  |  |  |

^a^ Base model is adjusted for age and sex
